# A knowledge curse: how knowledge can reduce human welfare

**DOI:** 10.1098/rsos.240358

**Published:** 2024-08-07

**Authors:** Kaushik Basu, Jörgen Weibull

**Affiliations:** ^1^ Department of Economics, Cornell University, Uris Hall 422, Ithaca, NY 14853, USA; ^2^ Department of Economics, Stockholm School of Economics, P.O. Box 6501, Stockholm 113 83, Sweden

**Keywords:** knowledge, game theory, evolution, welfare

## Abstract

Greater knowledge is always an advantage for a rational individual. However, this article shows that for a group of rational individuals greater knowledge can backfire, leading to a worse outcome for all. Surprisingly, this can happen even when new knowledge does not mean the discovery of a new action but simply provides a deeper understanding of the interaction at stake. More specifically, enhanced knowledge about the current state of nature may hinder cooperation among purely self-interested individuals. The paper describes this paradoxical possibility—a ‘knowledge curse’—and analyses the evolutionary process that occurs if, initially, only a few people have access to the greater knowledge. It concludes with a tentative comment on ways to avert this potential knowledge backlash.

## 1. Introduction

The advance of knowledge is normally seen as progress for humankind. Knowledge expands what human beings *can* achieve. As *Homo erectus* learned how to make and control fire, it expanded what they could eat and do for comfort in wintry days, and this made them better off. Greater knowledge expands the set of what is possible and, as long as we are rational, this can only be an advantage. Indeed, this is true for a Robinson Crusoe economy. A rational person on an island, discovering new truths, can only do better for herself.

Reality is more complicated. As Robert Oppenheimer realized, when watching the first detonation of a nuclear bomb on 16 July 1945, ‘We knew the world would not be the same.’ In this case, the scientific advance was the discovery of a new action, which meant that new strategies were now available. Clearly, the discovery of a new action can transform a game, which was otherwise a game of joint interests, into, for instance, a Prisoner’s Dilemma, to the detriment of all.

However, when knowledge does not expand anybody’s set of strategies, but instead enhances our understanding of the state of nature that together with our actions determines payoffs, it seems that it can only be to the benefit of humankind, or at least not lower human welfare. This article uncovers the finding that knowledge, even when that does not expand the set of available actions, can impact all of us negatively, what we refer to here as a *knowledge curse*.

One can see examples of the knowledge curse in domains where the behaviour of individuals is not in keeping with the collective interest, as happens with various common problems, such as precautionary behaviour during pandemics, like the coronavirus. Others wearing a mask is always good but to wear a mask oneself one must take into account the discomfort of wearing a mask. In such situations, not having full knowledge about the severity and nature of the contagion may result in greater collective welfare, which may break down if individuals have more refined information. For instance, in an epidemic with several strands of a virus, the expected private benefit of individual protection against the average strand, such as wearing a face mask, may exceed the private inconvenience or cost of taking such a precaution. Then all individuals will take the precautionary measures. For less harmful strands the private benefit of protection may fall short of the private cost and yet it may be in the public interest (weighing public health benefits against economic costs) that all take the precaution. Scientific progress in identifying different strands of the virus may then lead to a knowledge curse by way of disincentivizing individuals exposed to intermediate varieties.

Using a game-theoretic framework, this article also shows that if evolution occurs not over strategies, as in classical evolutionary game theory, pioneered by Maynard Smith and Price [1], but over player *types*, differentiated by knowledge or information, then knowledgeable players drive out the ignorant but may end up creating a world of diminished welfare, where welfare is defined as the average payoff in the population.

The insight that more information may be harmful has precursors in economics. One example is market failure owing to adverse selection, where one side of the market is better informed, a literature pioneered by Akerlof [2]. However, while in those situations some parties have ‘private information’, it has been shown that increased public information, available to all, can also be harmful in some cases. More precisely, the welfare effect of enhanced public information may be negative if agents also have independent private information, see [3–5]. This contrasts with the observations presented here, where public information can be harmful even in the absence of private information. Another case where enhanced public information may be harmful is insurance. The reason is that for risk-averse consumers, the existence of a perfectly competitive insurance market is welfare-enhancing. However, a scientific discovery that makes it possible for the insurance companies to identify those individuals who are more prone to a certain risk may cause these companies to deny them insurance. For seminal contributions, see [6,7]. Moreover, in game theory, it is known that in correlated equilibria, individual players may benefit from information being withheld, see Aumann [8].

Another observation of how knowledge may be harmful comes from psychology. An important topic concerns the proclivity of knowledgeable people to overestimate the knowledge of others. In the celebrated tapper–listener experiment by the Stanford psychology graduate student, Elizabeth Newton, human subjects were asked to pick a familiar song, like ‘Happy birthday’, and tap out the rhythm. The listeners were asked to guess what the tune was based on the morse-code like tapping sound that they heard. It was found that the tappers vastly overestimated the number of listeners who were able to guess the tune [9]. Interestingly, this propensity for the informed person to make mistakes stemming from the failure to judge the knowledge of others was called the ‘curse of knowledge’ in [10], who showed that ‘more information is not always better’. This has also been referred to as ‘hindsight bias’ and has been applied to many different contexts, see [11,12].

The knowledge curse that we are about to describe in this article is, despite similarities to some of the references mentioned above, a distinct idea. It is a paradoxical result since it appears rather unexpectedly in some environments of collective decision-making, and it is compatible with perfect rationality on the part of individuals.

## 2. Methods

Consider a two-person world, or a world of many who are matched pairwise and play the *Base Game* where one player chooses between the rows and the other columns.


$$\begin{array}{ccc} & A & B\\ A & 53,53 & 51,52\\ B & 52,51 & 50,50 \end{array}$$

The numbers represent expected payoffs that players obtain, with the row player’s payoff listed first and the column player’s listed second, for each of the four strategy combinations. Thus, if player 1 chooses *A* and 2 chooses *B*, 1 gets an expected payoff of 51 and 2 an expected payoff of 52. There may be variations in payoffs, but since they do not know when and why the variations occur there is not much they can do about this.

It is natural that players will settle into using the strictly dominant strategy *A*, which gives a higher expected payoff than strategy *B*, irrespective of what strategy the other player uses. As a consequence, they get an expected payoff of 53 each.

The assertion in §1, that the discovery of a new action can cause everybody’s welfare to drop can be illustrated with a simple example. Suppose that, in addition to actions *A* and *B*, there is discovery of a new action, *C*. Here is the *Expanded Base Game*, with action *C* for each player added in:


$$\begin{array}{cccc} & A & B & C\\ A & 53,53 & 51,52 & 0,55\\ B & 52,51 & 50,50 & 0,54\\ C & 55,0 & 54,0 & 1,1 \end{array}$$

What happens after this scientific advance? Both players will now choose their dominant strategy *C* and they will end up with a payoff of 1 each. There is, however, no surprise in this. The new action has converted the game into an extended Prisoner’s Dilemma.

What would be surprising is welfare collapsing with the advance of science even without the discovery of any new action. This counter-intuitive possibility is the focus of the present study. To examine it, consider a scientific advance. A Galileo Galilei comes along and discovers that if the moon is in phase 1, the game that humans play is the *Phase-1 Game* given below


$$\begin{array}{ccc} & A & B\\ A & 4,4 & 101,1\\ B & 1,101 & 98,98 \end{array}$$

That is, if the moon is in phase 1 and the two players choose action *A*, they will get payoff 4 each. And so on. Strategy *A* is still strictly dominant.

Galilei also discovers that if the moon is in phase 2, then the game that humans play is the following *Phase-2 Game* :


$$\begin{array}{ccc} & A & B\\ A & 102,102 & 1,103\\ B & 103,1 & 2,2 \end{array}$$

In this phase, strategy *A* is strictly dominated by strategy *B*, and if both players use this strategy, they each receive payoff 2.

Now note that the expected payoffs, when both phases of the moon are equally likely, are the ones given in the *Base Game* payoff bimatrix. That is, if, for instance, both choose *A*, each will half the time (when the moon is in phase 1) get 4 and half the time (phase 2) get 102, which makes for an expected payoff of 53.

Once the knowledge of the scientific breakthrough spreads through society, human income drops to an expected level of 3, the average of payoff 4 in phase 1 and payoff 2 in phase 2. This calamitous collapse, from 53 to 3, is brought about simply by the advance of knowledge.

It is not our claim that this will always happen, but the fact that it can is alarming enough. There is a lot of writing about the existential risks that human society faces. However, we tend to associate such risks with human folly, irrationality and lack of knowledge. What is summed up in the above parable is that in some interactions, perverse outcomes can be the result of science and rationality.

## 3. Results

### 3.1. General 2 × 2 games

To elaborate on this theme, consider a symmetric 2 × 2 game with payoff matrix for the row player


(3.1) $$\left[\begin{array}{cc} \pi_{11} & \pi_{12}\\ \pi_{21} & \pi_{22} \end{array}\right]$$

where *π*_11_ *> π*_21_ and *π*_12_ *> π*_22_ and the column player’s payoff is *π*_ji_ when the row player obtains *π*_ij_. Hence, pure strategy 1, previously called strategy *A*, strictly dominates pure strategy 2 (or *B*) for each player. The payoff entries in the matrix are the expected payoffs when the state of nature is unknown.

Suppose two possible states of nature that occur with probability *λ* and 1 – *λ*, respectively, where 0 *< λ< *1. The payoff matrices in these states are *π'* and *π*", respectively, where


(3.2) $$\left[\begin{array}{cc} \pi \prime_{11} & \pi \prime_{12}\\ \pi \prime_{21} & \pi \prime_{22} \end{array}\right]\;and\;\left[\begin{array}{cc} \pi \prime \prime_{11} & \pi \prime \prime_{12}\\ \pi \prime \prime_{21} & \pi \prime \prime_{22} \end{array}\right]$$

and *π*_ij_ = *λπ'*_ij_ + (1 – *λ*)*π"*_ij_ for all strategy combinations *i,j*∈{1,2}.

Assume strategy 1 strictly dominates strategy 2 also in *π'* (in state of nature 1), but that strategy 2 strictly dominates strategy 1 in *π"* (in state of nature 2). Formally, *π'*_11 _> *π'*_21,_ *π'*_12_ > *π'*_22,_ *π"*_11_ < *π"*_21_ and *π"*_12_ < *π"*_22_. All these conditions are met in the example in the preceding section; there *λ =* ½, *π*_11_ = (*π'*_11_ + *π"*_11_)/2 = 53, *π*_12_ = (*π'*_12_ + *π"*_12_)/2 = 51, and so on.^1^

In the present more general case, when both players are ignorant about the state of nature when choosing a strategy, they both expect payoff


(3.3) $$\pi \left(0\right)=\lambda \pi \prime_{11}+\left(1-\lambda \right)\pi \prime \prime_{11}.$$

Likewise, the payoff to each player in the first state of nature, if known by both, is *π'*_11_, and the payoff to each player in the second state of nature, if known by both, is *π"*_22_. Thus, the *ex-ante* expected payoff in a situation where both players know the state of nature when choosing a strategy is


(3.4) $$\pi \left(1\right)=\lambda \pi \prime_{11}+\left(1-\lambda \right)\pi \prime \prime_{22}.$$

As illustrated in §2, it is possible that *π* (0) > *π* (1), that is, players are better off if ignorant about the state of nature. In that example, we had *π* (0) = 53 *> π* (1) = 3. In other words, the *ex-ante* expected equilibrium outcome when both parties will get to know the state of nature before making their decisions is Pareto dominated by the *ex-ante* expected equilibrium outcome when they do not know the state of nature when making their decisions. We note that there is a knowledge curse, *π* (0) > *π* (1), if and only if *π"*_11_ > *π"*_22_.

### 3.2. A two-types model

The model so far was premised on the assumption that either both players know the state of nature, or neither does. In order to simplify the algebra, we henceforth assume *λ* = 1/2.^2^ Now suppose there is a population share *p*∈ [0,1] of knowledgeable individuals while other individuals are ignorant. Knowledgeable individuals are described as type 1, ignorant as type 0. Assume uniform random pairwise matching, that is, the conditional probability for one’s opponent’s type is independent of one’s own type. Let *ν*_0_ (*p*) be the expected payoff to individuals of type zero, the ignorant, and *ν*_1_(*p*) the payoff to knowledgeable individuals. Then


(3.5) $$v_{0}\left(p\right)=(1-p)\frac{\pi \prime_{11}\;+\;\pi \prime \prime_{11}\;}{2}+p\frac{\pi \prime_{11}\;+\;\pi \prime \prime_{12}\;}{2}$$

for all *p*, where $\frac{1}{2}$ (*π'*_11_ + *π"*_12_) is the expected payoff to an ignorant individual (always using strategy 1) when meeting a knowledgeable individual (who uses strategy 1 in state 1 and strategy 2 in state 2). Likewise,


(3.6) $$v_{1}\left(p\right)=(1-p)\frac{\pi \prime_{11}\;+\;\pi \prime \prime_{21}\;}{2}+p\frac{\pi \prime_{11}\;+\;\pi \prime \prime_{22}\;}{2}$$

for all *p*. By assumption, *π"*_21_ > *π"*_11_ and *π"*_22_ > *π"*_12._ Thus,


(3.7) $$v_{1}\left(p\right)-v_{0}\left(p\right)=(1-p)\frac{\pi \prime \prime_{21}\;+\;\pi \prime \prime_{11}\;}{2}+p\frac{\pi \prime \prime_{22}\;–\;\pi \prime \prime_{12}\;}{2}>0$$

for all *p*. In all population states, it is thus better to be knowledgeable than ignorant. We also note that for an ignorant individual, it is worse to meet a knowledgeable rather than ignorant individual if and only if *π"*_11_ > *π"*_12_.

In the *Base Game*, *Phase-1 Game* and *Phase-2 Game* in §2.1, the expected payoffs are steeply decreasing in the population share *p* of informed individuals:


(3.8) $$v_{0}\left(p\right)=\frac{1}{2}\left(106-101p\right)\;\text{a}nd\;v_{1}\left(p\right)=\frac{1}{2}\left(107-101p\right).$$

The difference *ν*_1_(*p*) – *ν*_0_(*p*) is thus 1*/*2 payoff unit for all *p*. In that example, individuals would thus have an incentive to inform themselves and become knowledgeable if and only if the cost of information is below 1*/*2. The end result would be a fully knowledgeable population, earning the low average payoff of 3. However, if the cost of information would exceed 1*/*2, then all individuals would have an incentive to remain uninformed in all population states. The end result would then be a population where all are ignorant of the state of nature but earn the high average payoff of 53.

The above calculations are based on the assumption that uninformed individuals, who do not know the current state of nature, use pure strategy 1. But is this always rational for them to do? That depends on the game payoffs, the population state, and on what they know. Suppose that a population share *p* of individuals are informed of the current state of nature and that the others are not. All informed individuals will then use pure strategy 1 in state of nature 1 and strategy 2 in state of nature 2. So the value of *p* does not matter for their strategy choice.

Now consider an uninformed individual. Suppose that she knows the two payoff matrices in (3.2) and the probability *λ* = 1/2 for state of nature 1. The uninformed individual may or may not know the population share *p* of informed individuals. Since she cannot condition her strategy choice on the state of nature, or, by assumption, on others’ behaviours (while in practice she might try to observe and imitate informed individuals), she has to stick to either pure strategy 1 or pure strategy 2, irrespective of the state of nature.

Suppose that all uniformed individuals always use pure strategy 1 and that the population state is *p*. Then the expected payoff to an uninformed individual is *ν*_0_(*p*), defined in equation (3.5). In order for this strategy choice to be optimal, the expected payoff *ν*_0_(*p*) has to be at least as high as the expected payoff had she instead always used pure strategy 2. It is easily verified that this condition holds for all values of *p* if and only if the payoff difference (*π'*_11_ *− π'*_21_) weakly exceeds the maximum of the two payoff differences (*π"*_21_ *− π"*_11_) and (*π"*_22_ *− π"*_12_). The condition simply requires that the payoff gain of using the right strategy in state 1, when meeting someone who also uses the right strategy for that state (and all others do), should be at least as large as the maximal payoff loss from using the wrong strategy in state 2, when meeting someone who either uses pure strategy 1 (an uninformed individual) or 2 (an informed individual) in that state of nature.

In our lead example, the *Base Game*, *Phase-1 Game* and *Phase-2 Game* in §2, the first payoff difference is 3 and the second 1. So in this example, and in many others, it does not matter for uniformed individuals what the population state is; strategy 1 is optimal.

But are state-ignorant individuals who know the payoff matrices and the probabilities for the states of nature plausible? Arguably yes. For example, individuals may well be ignorant of the current strand of a virus in an epidemic but may still know the payoff matrices under different strands, and the probabilities for these strands.

### 3.3. Evolutionary selection

**Figure 1.**
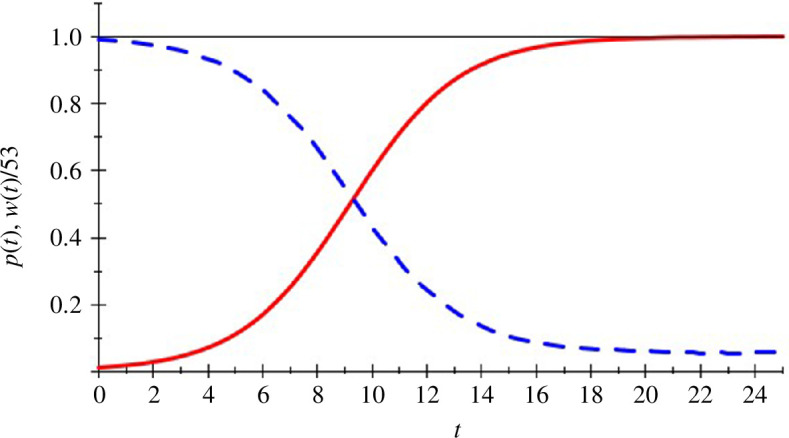
Evolution over time of the population state and welfare. The solid curve shows how the population share, *p*(*t*), of knowledgeable individuals evolves over time *t* according to a replicator dynamic (see e.g. [13]) applied to the example in §2.1, with initially 1% knowledgeable individuals. More precisely, we used the two-traits replicator dynamic $\overset{˙}{p}=(\nu_{1}(p)\;–\;\nu_{0}(p))(1\;-\;p)p$ with initial condition *p*(0) = *p* ^0^. The dashed curve shows how welfare, *w*(*t*), defined as the average payoff in the population, evolves over time. (To fit on the vertical axis, welfare is divided by 53.) The population share of knowledgeable individuals grows over time and tends asymptotically to 1, and welfare declines from its initial value close to 53 and tends asymptotically to 3.

Since knowledgeable individuals obtain a higher expected payoff than those who are ignorant, evolutionary selection of types, if driven by payoffs, would favour knowledgeable individuals. In the spirit of John Maynard Smith’s and George Price’s definition of an evolutionarily stable *strategy* [1], one could say that to be knowledgeable is an ‘evolutionarily stable *trait*’ in the sense that under uniform random pairwise matching knowledgeable individuals earn a strictly higher payoff than those who are ignorant. Moreover, this evolutionary stability of being knowledgeable is strong, since the payoff difference is positive in *every* population state, and not just when ignorant individuals make up a small minority (‘rare mutants’ in Maynard Smith’s and Price’s definition of evolutionary stable strategies). In the long run, in an evolutionary selection dynamic that favours traits that give higher payoffs, ignorance will be asymptotically wiped out from the population (from any initial population with a positive share of knowledgeable individuals). That knowledge provides an evolutionary advantage to its carriers, and will over time drive out ignorance, is not a surprising result. What is surprising is that knowledge, by driving out ignorance, ends up creating a society that has lower welfare than would have happened if the knowledge in question had not arrived. See figure 1 below.

The above considerations were based on the assumption that knowledge about the scientific discovery is costless. If there were a positive cost associated with being well-informed (such as the need to get college education), then a positive population share of ignorant individuals may well persist even in the long run.

The evolutionary calculations behind figure 1 are based on strong assumptions, of which two will be briefly discussed here. The first assumption is the uniformity of the random pairwise matching. However, the replicator dynamic can easily be generalized to allow for assortative random matching, whereby knowledgeable individuals are more likely to be matched with other knowledgeable individuals than pure proportionality would suggest. The source of such assortativity may be cultural, educational or geographical. To analyse the effect of assortativity, let *P*(*p*) = *a *+ (1 – *a*)*p* be the probability in population state *p* that a knowledgeable individual’s match is also knowledgeable. Here *a* is a parameter between zero and one, the *degree of assortativity*. At zero degree of assortativity, *P*(*p*) = *p* in all population states, as in the standard replicator dynamic, while at unit degree of assortativity, knowledgeable individuals are always matched with each other. Likewise, let *Q*(*p*) be the probability in population state *p* that an ignorant individual’s match is a knowledgeable individual. It is then necessary that Q(*p*) = (1 *– a*)*p* in all population states *p*, see [14].

By a straightforward generalization of the standard replicator dynamic one then finds that for low enough degrees of assortativity, the population state *p* = 1 (only knowledgeable individuals) is globally stable, just as in the replicator dynamic. Conversely, for high enough degrees of assortativity, the population state *p* = 0 (only ignorant individuals) is globally stable. This is because if there is a knowledge curse, *π* (0) > *π* (1), then ignorant individuals, matched with each other, thrive. For intermediate degrees of assortativity (depending on payoffs) there may exist a globally stable *interior* population state, one with 0 < *p* < 1. In such a population state, knowledgeable and ignorant individuals coexist in fixed proportions and have exactly the same expected payoff.^3^

The second strong assumption is that the population share of knowledgeable individuals can diminish over time. This is possible in intergenerational dynamics, where knowledgeable parents may have ignorant children. However, in socio-cultural evolution, a knowledgeable individual can hardly become ignorant. It then seems more reasonable to assume that the population share of knowledgeable individuals cannot diminish, at least not in the short run. This will not change the above results for low degrees of assortativity, but for intermediate and higher degrees of assortativity the analyst may find whole intervals of Lyapunov stable population states that are not asymptotically stable.^4^ For such degrees of assortativity, the population can thus get stuck in wide ranges of mixed-population states.

### 3.4. General finite games

To elaborate on the knowledge curse in a more general game-theoretic setting, consider any finite *n*-player normal-form game in which payoffs are state dependent and there are finitely many possible states *ω *∈ Ω that materialize with positive probabilities *µ*(*ω*). For any pure-strategy profile $s=(s_{1},...,s_{n})\in S=\times \;{,}_{i=1}^{n}S_{i}$ , where $S_{i}$ is the set of pure strategies available to player i∈I={1,...,n} , and any state *ω *∈ Ω, let $\pi_{i}^{\omega }(s)$ be the payoff to player *i*, and let ${\overset{-}{\pi }}_{i}(s)$ be the player’s expected payoff:


(3.9) $${\bar{\pi }}_{i}\left(s\right)=\sum_{\omega \;\in \;\Omega }\mu \left(\omega \right)\pi_{i}^{\omega }\left(s\right).$$

Denote by *G_ω_* the game in state *ω *∈ Ω, that is, with payoffs $\pi_{i}^{\omega }(s)$ , and let $\overset{-}{G}$ be the game with payoffs ${\overset{-}{\pi }}_{i}(s)$ . All these games have the same set *I* of players, and each player’s pure-strategy set $S_{i}$ is the same in all games.

Suppose that $\overset{-}{G}$ has a unique ‘solution’ s^*^ ∈ *S*. By a ‘solution’ is here meant any prediction the analyst makes for how the game will be played. It may, for example, consist of one strictly dominant strategy for each player, as in the above numerical example, or be the unique Nash equilibrium of the game in question, or some other solution concept that the analyst deems appropriate for the application at hand. Suppose, moreover, that each game *G_ω_* also has a unique solution, $\hat{s}$ *^ω^* ∈ *S*, and that this differs from s* in some states *ω *∈ Ω. If a scientific discovery is made that enables costless identification of the current state for all players *i *∈ *I*, then these will all deviate from s* to $\hat{s}$ *^ω^* in such a state *ω *∈ Ω. The discovery leads to lower expected payoffs for all players if and only if


(3.10) $$\sum_{\omega \;\in \;\Omega }\mu \left(\omega \right)\pi_{i}^{\omega }\left({\hat{s}}^{\omega }\right)<{\bar{\pi }}_{i}\left(s^{*}\right)$$

for all players *i*. These inequalities hold in the numerical lead example, and (3.10), which is a generalization of *π* (0) > *π* (1), can be viewed as a definition of a ‘knowledge curse’, a situation in which new knowledge, through its effect on individuals’ behaviour, reduces expected welfare in society.

We note that the knowledge curse may strike in many kinds of games, including games involving conflict, such as the well-known Hawk–Dove game (also called ‘Game of Chicken’ and ‘Snowdrift Game’).^5^ This is a symmetric two-player game representing a conflict over a resource (such as food or money) of positive value *v*. Each player has to choose between ‘fight’ (Hawk) or ‘yield’ (Dove). If both choose fight, they have equal probability of winning. The winner obtains payoff *v* and the loser incurs a payoff loss or cost *c*, where 0 < *v *< c. If one player chooses fight and the other yield, then the first obtains *v* and the second obtains 0. This game has a unique evolutionarily stable strategy, namely, to fight with probability *v/c*. If both players use this strategy, then the expected payoff to each player is (1 − *v*/*c*)*v*/2. If one takes this to be the solution of the Hawk–Dove game, then the knowledge curse strikes if, for example, *v* = 1 and *c* is either 2 or 4, with equal probability for both values. Under ignorance, the expected cost is thus 3 and the expected payoff 1/3. Under knowledge of the current value of *c*, the *ex-ante* expected payoff is (1/4 + 3/8)/2 = 5/16 < 1/3.

## 4. Discussion

From the basic result presented in this article, it is possible to venture out in different directions. One could, for instance, ask what would happen if the scenario would change from playing the game once at each random matching to repeated play of the game at each matching. Under ignorance, not much would change in the *Base Game* in §2. Under statistically independent and identically distributed payoffs each period, each player would be able to guarantee him- or herself an expected payoff of at least 51 per period and obtain at most 53.^6^ However, if all individuals would know the current state in every period, matters would change. As we know from the folk theorems for repeated games, many other outcomes would then be possible, with payoffs ranging from 4 to 98 in phase 1 and from 2 to 102 in phase 2. One possibility is to play in each period just as in the one-shot game. The set of potential outcomes would depend on the discount factor that individuals place on future payoffs (their ‘patience’), the probability and duration of continued play in the matched pair, and the nature of the stochastic process that determines period payoffs.^7^

We close with some remarks on pre-emptive policy to avert the knowledge curse. Policymaking without knowing the full contours of the problem is not easy. The aim is to have prior agreements about what to do if and when we confront a ‘knowledge curse’.

Suppose that *π* (0) *> π* (1), that is, welfare is highest if all individuals are ignorant about the true state of nature. A benevolent social planner would prefer to keep all individuals ignorant, or to restrict their actions. Such restriction might be achieved by mandating the socially desirable act when individuals lack the personal incentive to undertake it, or by taxing socially undesirable acts.

Societies have taken these kinds of actions in the past. The drafting of constitutions that apply well into the unknown future is one such normative action. Such pre-emptive laws have conferred large benefits to humankind.

Idealistic though this may sound, we can think of developing norms that can kick in when a new danger emerges. In traditional economics, we take people’s preferences as exogenous. There is widespread recognition that the game of managing climate change is a kind of Prisoner’s Dilemma and if each of us lives by our own self-interest, we will end up with an environmental calamity. As a result, we are learning that in contexts pertaining to the environment, we must not act to maximize our individual payoffs. The capacity to rethink our individual choices in terms of the common good is not as impossible as traditional economics makes it out to be. Indeed, if individuals are sufficiently morally motivated ‘to do the right thing’, then the knowledge curse dissolves; more knowledge is then always beneficial. The reason is that then knowledgeable individuals will adapt their actions to the current state of nature in such a way that it benefits the common good (e.g. [19]). Therein lies the hope that we may be able to avoid some of the dangers that the paper draws attention to without having to heavily tax or otherwise punish socially undesirable acts.

The belief that whatever happens because of *human* choice can be prevented is not valid. Game theory is a reminder that when a group of people interact, and each person is held responsible for her actions, we cannot always carry this logic over to the group, holding the group responsible for the combined action profile that the group ‘chooses’, see [20]. As analysts, we have to design rules to guard against the adversities. This article is a nudge to explore new normative grounds.

## Data Availability

This paper contains no data. It is purely theoretical.

## Footnotes

^1^
We here use the word ‘strategy’ in an informal way. In formal game theory, a player’s strategy is a rule that assigns an action to each decision problem (information set) that the player may face. In that terminology, ignorant players have only two pure strategies, actions A and B, respectively, while knowledgeable players have four: to take action A (B) in both states of nature, to take action A (B) in state 1 and action B (A) in state 2. Since this is intuitively obvious, we have chosen to avoid this cumbersome terminology.

^2^
The results are qualitatively the same for any *λ* that lies strictly between zero and one.

^3^
For this to happen, it is necessary and sufficient that the following two inequalities hold: 
*π"*_11_ < *aπ"*_22_ + (1 – *a*)*π"*_21_ and *π"*_22_ < *aπʹ*_11_ + (1 – *a*)*π"*_12_ .

^4^
A population state is *Lyapunov stable* if small perturbations do not initiate movement away from the state. Technically, it requires that every neighbourhood of the state contains a sub-neighbourhood such that all dynamic trajectories that start in the sub-neighbourhood remain forever in the given neighbourhood.

^5^
It was introduced in the biology literature in Ref. [1].

^6^
Note that under autocorrelation in the stochastic payoff process, even ignorant individuals could start to see temporal patterns, and this could invoke a knowledge curse also over them.

^7^
Under infinite repetition with random changes of payoffs, the game played is a so-called stochastic game, for which there exist theoretical results that generalize the folk theorems for repeated games. Relevant references for the present study are [15–18].
